# The impact of climate change and natural disasters on the development of post traumatic stress disorder in child and adolescent population

**DOI:** 10.1192/j.eurpsy.2023.635

**Published:** 2023-07-19

**Authors:** B. Ghosh Dastidar

**Affiliations:** Psychiatry, Imperial College NHS Trust CNWL, London, United Kingdom

## Abstract

**Introduction:**

Major traumatic natural disasters have occurred worldwide. Post-traumatic stress disorder (PTSD) has been the most common psychiatric disorder discussed by the studies addressing the psychological sequelae of adolescents after traumatic natural disasters. In this study we have studied the impacts of natural disaster yash cyclone that took place in West Bengal on the development of PTSD; factors related to the development of PTSD; predisposing, precipitating, and perpetuating factors related to the development of PTSD.

**Objectives:**

To assess the incidence and prevalence of ptsd amongst survivors of natural disaster yash cyclone in a rural hamlet of West Bengal.

**Methods:**

In this study ,200 survivors from Yash cyclone who belonged to Child and Adolescent age group were randomly selected .PCL 5 Scale was used to collect data and assess the incidence and prevalence of PTSD , standardized Bengali versions of the questionnaire was used in our study.

**Results:**

There is statistical correlation between post traumatic stress disorder and subjects exposed to climate change events such as cyclone Yash.

Initial research suggests that a PCL-5 cut-off score between 31-33 is indicative of probable PTSD across samples.

In our study the mean pcl 5 value from the data assessment is 70.67 with standard deviation of 4.61.

Further assessment by linear regression analyses shows that female subjects are more prone to post traumatic stress disorder and higher income groups are more susceptible to ptsd.

As shown by higher values as per the pcl 5 scale.

**Conclusions:**

Our study clearly demonstrates the impact of climate change and natural disasters on the mental health status of people living in disaster prone areas especially the child and adolescent population. Our study group was child and adolescent population between 10 to 15 years.

The psychologist and volunteers had to collect data in disaster affected zone , yet they collected data which gave a clear cut findings and a very clear statement on climate change and mental health. The values are very high and consistent in most subjects across all twenty domains.

It is our opinion that Mental health support should be provided for all victims of climate change and natural disaster calamities such as cyclone and earthquake.

**Disclosure of Interest:**

None Declared

